# DNA-mediated proteolysis by neutrophil elastase enhances binding activities of the HMGB1 protein

**DOI:** 10.1016/j.jbc.2022.102577

**Published:** 2022-10-08

**Authors:** Xi Wang, Marlen Mayorga-Flores, Karina G. Bien, Aaron O. Bailey, Junji Iwahara

**Affiliations:** Department of Biochemistry & Molecular Biology, Sealy Center for Structural Biology & Molecular Biophysics, University of Texas Medical Branch, Galveston, Texas, USA

**Keywords:** DNA–protein interaction, fluorescence anisotropy, NMR, protein processing, protease, toll-like receptor 4, DAMP, damage-associated molecular pattern, FAM, fluorescein amidite, FL, full-length, NET, neutrophil extracellular trap, SPR, surface plasmon resonance

## Abstract

Neutrophil extracellular traps (NETs) are produced through ejection of genomic DNA by neutrophils into extracellular space and serve as a weapon to fight against pathogens. Neutrophil elastase, a serine protease loaded on NETs, attacks and kills pathogens, while extracellular high-mobility-group-box-1 (HMGB1) protein serves as a danger signal to other cells. How the action of these factors is coordinated as part of the innate immune response is not fully understood. In this article, using biochemical and biophysical approaches, we demonstrate that DNA mediates specific proteolysis of HMGB1 by neutrophil elastase and that the proteolytic processing remarkably enhances binding activities of extracellular HMGB1. Through the DNA-mediated proteolysis of HMGB1 by neutrophil elastase, the negatively charged segment containing D/E repeats is removed from HMGB1. This proteolytic removal of the C-terminal tail causes a substantial increase in binding activities of HMGB1 because the D/E repeats are crucial for dynamic autoinhibition *via* electrostatic interactions. Our data on the oxidized HMGB1 (*i.e.*, ‘disulfide HMGB1’) protein show that the truncation substantially increases HMGB1’s affinities for the toll-like receptor TLR4•MD-2 complex, DNA G-quadruplex, and the Holliday junction DNA structure. The DNA-mediated proteolysis of HMGB1 by neutrophil elastase in NETs may promote the function of extracellular HMGB1 as a damage-associated molecular pattern that triggers the innate immune response of nearby cells.

In mammalian tissues, DNA in the extracellular space may represent a danger associated with infection or tissue damage (1, 2). Infecting bacteria often thrive in biofilms that utilize extracellular DNA to stabilize colonies through a matrix involving such atypical DNA structures as Holliday junction, G-quadruplex, and Z-form (3, 4, 5). In addition to extracellular DNA of pathogens, necrosis of host cells at a damaged tissue also causes a release of DNA fragments to extracellular space (6, 7). DNA fragments and some DNA-binding proteins released to extracellular space serve as damage-associated molecular patterns (DAMPs) that activate some cell surface receptors and initiate innate immune response of nearby cells (8, 9).

The high-mobility-group-box-1 (HMGB1) protein is one of the most potent DAMPs in mammals and plays several important roles in innate immunity involving extracellular DNA. Although HMGB1 typically serves as a chromatin-associated protein that bends DNA (10, 11), HMGB1 is released to extracellular space not only passively from dying cells but also actively from platelets and some healthy cells (*e.g.*, macrophages, astrocytes, and dendritic cells) (12, 13). Extracellular HMGB1 can destabilize scaffold DNA matrix of bacterial biofilm (14). Extracellular HMGB1 is also involved in endocytosis of extracellular DNA into cells (15, 16). Interactions between extracellular HMGB1 and an immune checkpoint receptor TIM-3 prevent the DNA uptake process (15, 17). HMGB1 activates toll-like receptor 2 and 4 (TLR2 and TLR4) for innate immune response (18, 19). Through interactions with DNA containing unmethylated CpG dinucleotides, HMGB1 also activates TLR9 (20, 21). HMGB1 can shuttle from the extracellular space to endosomes and cytosol (15, 16, 22). In the cytoplasm, the ability of HMGB1 to sharply bend DNA enhances the interactions between cyclic GMP-AMP synthase (cGAS) and DNA fragments, which activates the cGAS-STING pathway for innate immune response (23).

Extracellular HMGB1 stimulates neutrophils, the most abundant innate immune effector cells of the human immune system, and can ultimately induce ejection of their chromosomal DNA, forming neutrophil extracellular traps (NETs) (24, 25). Casting a web-like structure, NETs trap and kill pathogens (26, 27). One of the major weapons for NETs to kill pathogens is neutrophil elastase, a serine protease bound to DNA of NETs (26). DNA-bound neutrophil elastase retains its proteolytic activity and attacks pathogenic proteins as well as some host proteins such as plasminogen (26, 28). Because both neutrophil elastase and HMGB1 are capable of binding to DNA and are abundant in NETs (28, 29), it seems likely that HMGB1 encounters neutrophil elastase within NETs.

Several previous studies have reported that HMGB1 can be processed by immune-related proteases: namely, caspase 1, cathepsin G, complement factor C1s, matrix metalloproteinase 3, and neutrophil elastase (30, 31, 32). Using experimental conditions without DNA, Sowinska *et al.* (31) biochemically showed that neutrophil elastase can cleave HMGB1 at several different sites. However, we should point out that DNA of NETs may impact the site specificity and catalytic efficiency in the proteolysis of HMGB1 by neutrophil elastase. DNA interacting with HMGB1 may protect the DNA-binding interfaces of HMGB1 from the attack of proteases while noninterfacial regions remain unprotected and cleavable. This effect would increase the site specificity in the proteolysis. Colocalization on DNA may also increase the chance for HMGB1 to encounter with neutrophil elastase and thereby enhance the kinetic efficiency in the proteolytic cleavage of HMGB1.

In this article, we demonstrate that DNA enhances both efficiency and site specificity in the proteolytic cleavage of HMGB1 by neutrophil elastase. HMGB1 is a redox sensitive protein and forms a disulfide bond between Cys23 and Cys45 in extracellular space (33, 34, 35). The oxidized HMGB1 protein (*i.e.*, often referred to as ‘disulfide HMGB1’) was used in our current biochemical and biophysical experiments because this form of HMGB1 is more relevant to the extracellular function than the reduced HMGB1 (‘all-thiol HMGB1’). We show that the DNA-mediated proteolysis of disulfide HMGB1 occurs specifically at the sites (mainly V176) between the B-box domain and the C-terminal D/E repeats. Importantly, this proteolysis causes a remarkable increase in the binding affinities of disulfide HMGB1 for G-quadruplex DNA, Holliday junction DNA, and TLR4•MD-2 complex. Therefore, the DNA-mediated proteolysis of HMGB1 by neutrophil elastase in NETs may play an important role in inflammation.

## Results

### DNA-mediated proteolysis of HMGB1 by neutrophil elastase

**Figure 1:**
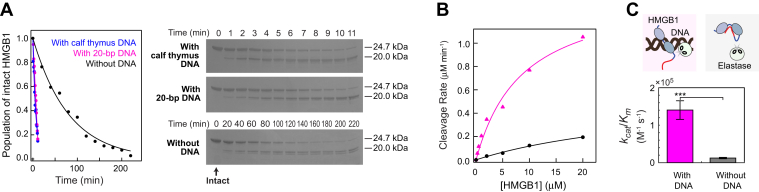
**DNA-mediated proteolysis of HMGB1 by neutrophil elastase.***A*, examples of reaction time course data and SDS-PAGE images showing the proteolysis of HMGB1 by neutrophil elastase in the presence and absence of DNA. For each case, the concentrations of disulfide HMGB1 and neutrophil elastase were 10 μM and 20 nM, respectively. The concentration of sonicated calf thymus DNA was estimated to be 400 μM base pairs. The concentration of the 20 bp DNA duplex was 20 μM. Because of different reaction rates, the time samplings for the SDS-PAGE data for the reactions without DNA were different. Entire gel images are shown in Fig. S1 in the Supporting information. *B*, reaction rates as initial velocities (36) for the cleavage of HMGB1 at various concentrations in the presence (*magenta*) and absence (*black*) of 20 bp DNA (20 μM). *C*, catalytic efficiencies (*k*_cat_/*K*_*m*_) for the proteolysis of HMGB1 by neutrophil elastase in the presence and absence of DNA. Individual values of the Michaelis–Menten parameters *K*_*m*_ and *k*_cat_ are indicated in Table 1. The *k*_cat_ and *K*_*m*_ parameters were determined through nonlinear least squares fitting to the data shown in Figure 1*B*. ∗∗∗*p* < 0.001.

Based on what is described previously, we hypothesized that DNA could impact the proteolysis of HMGB1 by neutrophil elastase. To test this hypothesis, HMGB1 proteins were processed by neutrophil elastase in the presence and absence of DNA. Figure 1*A* shows the Coomassie-stained SDS-PAGE gels for the reaction mixtures at some different time points. In the presence of sonicated calf thymus DNA (400 μM base pairs), the proteolysis of HMGB1 by neutrophil elastase was fast. A major product from the reaction was observed as early as in 1 min. A similar result was observed in the presence of 20 μM linear 20 bp DNA (corresponding to 400 μM base pairs). By contrast, the reaction in the absence of DNA was far slower and two major products and subsequently additional products were observed over hours (see Fig. S1 in the Supporting information). These data clearly show that DNA enhances the efficiency and site specificity in the proteolytic cleavage of HMGB1 by neutrophil elastase.

Through further experiments, we investigated the enzyme kinetics of the DNA-mediated proteolysis. Because the molarity of sonicated calf thymus DNA cannot be clearly defined, we used the 20 bp DNA duplex for the quantitative investigations of the proteolysis. Through densitometry of SDS-PAGE images, we quantitatively analyzed the time course of the proteolysis kinetics. We measured the reaction rates as initial velocities (36) for the proteolysis of HMGB1 at various concentrations in the presence and absence of the 20 bp DNA duplex. From the HMGB1 concentration dependence of the cleavage rate (Fig. 1*B*), we determined the Michaelis–Menten steady-state constant *K*_*m*_ and the catalytic rate constant *k*_cat_. Table 1 shows the values of these Michaelis–Menten parameters for the reaction in the presence and absence of DNA. The ratio *k*_cat_/*K*_*m*_ corresponds to a second order rate constant that represents the catalytic efficiency of an enzymatic reaction (36). As shown in Figure 1*C*, the catalytic efficiency of HMGB1 proteolysis by neutrophil elastase is increased by as much as ∼11-fold in the presence of DNA.

**Table 1:** Michaelis–Menten parameters *K*_*m*_ and *k*_cat_ for the proteolysis of HMGB1 by neutrophil elastase in the presence and absence of 20 bp DNA^a^

Parameters	With DNA^b^	Without DNA
*K*_*m*_ (M)	(8.7 ± 2.2) × 10^−6^	(39 ± 11) × 10^−6^
*k*_cat_ (s^−1^)	1.2 ± 0.1	0.48 ± 0.1

a Buffer conditions were 10 mM potassium phosphate (pH 7.4), 100 mM NaCl at room temperature. The uncertainties were estimated at a confidence interval of 68%.

b 20 μM 20 bp DNA with a sequence of CTCTGGACCTTCCTTTCTTC.

The data of *K*_*m*_ and *k*_cat_ (Table 1) provide mechanistic insight into how DNA promotes the HMGB1 cleavage by neutrophil elastase. The presence of DNA caused a significant decrease in the *K*_*m*_ constant by a factor of 4.4. This suggests that DNA increases the probability of encounter between neutrophil elastase and HMGB1, presumably through a transient ternary complex formation. DNA can also release the intramolecular interactions involving the C-terminal tail within HMGB1 (37). Compared to the kinetic data for cleavage of peptide substrates by neutrophil elastase in the literature (38, 39), our *k*_cat_ values are comparable, whereas the *K*_*m*_ value for the reaction in the presence of DNA is far smaller than those reported for the peptide cleavage by neutrophil elastase. These data support the notion that the decrease in *K*_*m*_ is more important for the DNA-mediated proteolysis by neutrophil elastase. However, we should point out that the *k*_cat_ constant also becomes larger in the presence of DNA. The crystal structures of neutrophil elastase show that the active site residue, Ser195, of this serine protease is located apart from the highly positively charged surface (Fig. S2 in the Supporting information). It is likely that neutrophil elastase uses the positively charged surface to interact with DNA. The DNA binding might allosterically affect the active site and thereby improve the catalytic rate *k*_cat_. Interestingly, a HMGB1 variant lacking the D/E repeats but retaining the cleavage site exhibited a slower proteolysis by neutrophil elastase (Fig. S3 in the Supporting information), which suggests that the positively charged surface of the neutrophil elastase may also contribute to substrate specificity. Thus, electrostatic interactions appear to play a role in the efficiency and specificity of HMGB1 cleavage by neutrophil elastase.

### Δ39 is the major product of DNA-mediated proteolysis

**Figure 2:**
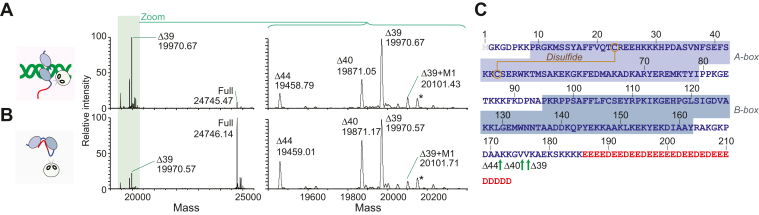
**LC-MS intact mass-based identification of the cleavage sites for the DNA-mediated proteolysis of disulfide HMGB1 by neutrophil elastase.***A* and *B*, deconvolved mass spectra produced for the reaction mixtures in the presence (panel *A*) and absence (panel *B*) of 20 bp DNA. The regions for the reaction products and the original disulfide HMGB1 protein are shown. In the presence of DNA, Δ39 was the major cleavage product and minor cleavage products were Δ40 and Δ44. The peaks indicated by “Δ39 + M1” correspond to the Δ39 product of the HMGB1 protein retaining the initial methionine M1. The peaks indicated by asterisks correspond to the Δ39 product of the gluconoylated HMGB1 lacking M1. The minor species with M1 or gluconoyl modification (73) were also observed for mass spectra recorded for the original proteins (see Fig. S4 in the Supporting information). For more detailed information about the species identified by LC-MS, see Table S1 in the Supporting information. *C*, amino acid sequence of HMGB1 and the identified cleavage sites (indicated by the *green arrows*).

Previous studies on substrate specificity of neutrophil elastase suggested that this serine protease exhibits only weak sequence specificity (40, 41). This ambiguity makes it challenging to predict the cleavage sites on HMGB1 from its amino acid sequence alone. We used LC-MS to analyze the intact mass profiles of disulfide HMGB1 samples treated with neutrophil elastase in the presence and absence of DNA. Deconvolution results of neutrophil elastase-treated full-length (FL) HMGB1 in the presence of DNA provide high mass accuracy identification of two major species Δ39 (∼60%) and Δ40 (∼30%), which lack C-terminal 39 and 40 residues, respectively, and a minor species corresponding to Δ44 (∼10%), which lacks C-terminal 44 residues (Fig. 2; see also Table S1 in the Supporting information), consistent with the major and minor bands present in the SDS-PAGE results. We confirmed the identities of these peaks through intact mass analysis of recombinant FL HMGB1 and Δ40 variant control samples, each of which was separately expressed and purified by chromatographic methods. These data indicate that the major product of the DNA-mediated proteolysis of HMGB1 by neutrophil elastase is Δ39.

We also conducted the same LC-MS analysis for reaction mixtures for the HMGB1 proteolysis in the absence of DNA. The data show the same species: Δ39 (∼45%), Δ40 (∼30%), and Δ44 (∼25%). However, compared to the DNA-mediated proteolysis, the preference for Δ39 was weaker and the relative population of Δ44 was higher. In fact, in the SDS-PAGE images shown in Figure 1*A*, the relative intensity of the band of the Δ44 product appears stronger for the reaction in the absence of DNA. These results suggest that the proteolysis of HMGB1 by neutrophil elastase in the absence of DNA is not only slower but also less selective.

### Impact of the HMGB1 truncation on affinities for G-quadruplex and Holliday junction

**Figure 3:**
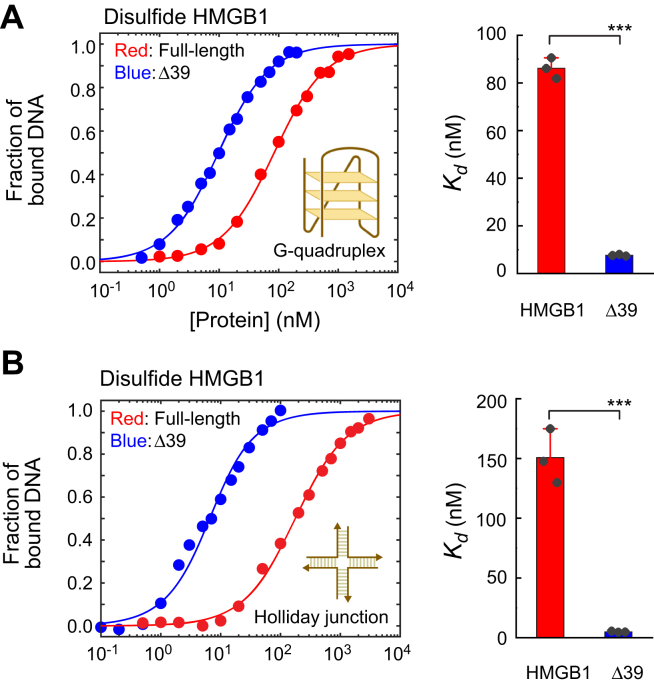
**The main product of HMGB1 proteolysis by neutrophil elastase exhibits substantially stronger binding affinity for DNA G-quadruplex and Holliday junction.** Fluorescence anisotropy-based binding assays were used to measure affinities for a G-quadruplex and a Holliday junction. *A*, binding isotherm data for the interactions of the intact disulfide HMGB1 and Δ39 proteins with FAM-labeled G-quadruplex DNA (32-mer). Corresponding data for the Δ40 and Δ44 products are shown in Fig. S5 in the Supporting information. *B*, binding isotherm data for the interactions of the intact disulfide HMGB1 and Δ39 proteins with FAM-labeled Holliday junction. For each *K*_d_, error bars represent the SDs for three replicates. ∗∗∗*p* < 0.001.

Since Δ39 is the major product of the DNA-mediated proteolysis of HMGB1 by neutrophil elastase, we prepared a recombinant Δ39 variant protein and compared its molecular properties to those of the FL disulfide HMGB1 protein. Through fluorescence experiments, we examined the extent to which the proteolytic truncation impacts the DNA-binding affinities of disulfide HMGB1 for a DNA G-quadruplex named 32G (42) and a DNA Holliday junction named J1 (43). They were chosen for the current investigation because extracellular DNA of some bacterial biofilms contains G-quadruplex DNA (5) and Holliday junctions (4, 14) and HMGB1 is known to strongly interact with these atypical DNA structures (14, 42, 44, 45, 46, 47). Figure 3 shows the binding data from the fluorescence anisotropy–based assays on association with the DNA molecules for the FL and the Δ39 proteins. For each protein, we determined the dissociation constant, *K*_d_, for the complex with the G-quadruplex (Fig. 3*A*) and for the complex with the Holliday junction (Fig. 3*B*). For both G-quadruplex and Holliday junction, the affinity of the Δ39 product was remarkably higher than that of FL HMGB1. For 32G, we also conducted the affinity measurements for the other cleavage products Δ40 and Δ44 and found equally strong affinity compared with Δ39 (Fig. S5 in the Supporting information). Thus, the DNA-mediated proteolysis by neutrophil elastase promotes the binding affinities of HMGB1 for the G-quadruplex DNA as well as for the Holliday junction DNA.

### Impact of the HMGB1 truncation on affinity for TLR4•MD-2

**Figure 4:**
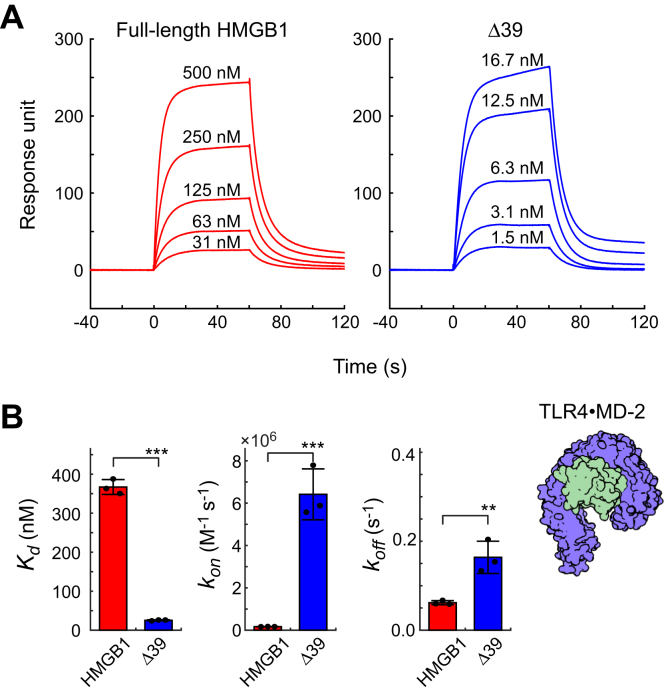
**Surface plasmon resonance (SPR) data showing that the affinity of the main proteolysis product Δ39 for TLR4•MD-2 complex is higher than the affinity of the intact HMGB1 protein.***A*, SPR sensorgram data on disulfide HMGB1 and its Δ39 variant for their interactions with TLR4•MD-2 complex. *B*, comparison of the dissociation constants (*K*_*d*_), the association rate constants (*k*_*on*_), and the dissociation rate constants (*k*_*off*_) for the intact disulfide HMGB1 protein and its Δ39 variant. Error bars represent the SDs from three replicates. ∗∗*p* < 0.01; ∗∗∗*p* < 0.001.

TLR4 is one of the most important HMGB1 receptors in innate immune response to DAMPs (48). Surface plasmon resonance (SPR)–based assays for the binding of HMGB1 to TLR4•MD-2 complex have been well established (19, 49). To examine the extent to which the removal of the C-terminal tail affects HMGB1’s interaction with TLR4•MD-2 complex, we conducted the SPR assays for the FL HMGB1 protein and the Δ39 product. The obtained SPR data are shown in Figure 4. Compared to the FL HMGB1 protein, the Δ39 product caused a large increase in response units at lower concentrations in the SPR sensorgram data, reflecting Δ39’s stronger affinity. The dissociation constants (*K*_*d*_) determined from the SPR sensorgram data were 367 ± 19 nM for the FL disulfide HMGB1 protein and 25 ± 1 nM for Δ39. Thus, the affinity of Δ39 for TLR4•MD-2 complex was stronger than that of the FL HMGB1 protein by a factor of 14. Interestingly, as indicated by the associate rate constants (*k*_*on*_) and the dissociation constants (*k*_*off*_), the stronger affinity of Δ39 is caused by faster association rather than the slower dissociation. Our data show that the DNA-mediated proteolysis of HMGB1 by neutrophil elastase enhances the binding affinity of HMGB1 for the TLR4•MD-2 complex.

### Intramolecular interactions of disulfide HMGB1

The affinity enhancement *via* the truncation of HMGB1 by neutrophil elastase can be explained in terms of autoinhibition of FL HMGB1. The C-terminal 30 residues of HMGB1 are highly negatively charged D/E repeats comprising of either aspartate (D) or glutamate (E) (see Fig. 2*C*). For all-thiol HMGB1, intramolecular interactions between the negatively charged D/E repeats and the positively charged regions (*e.g.*, A-box and B-box domains) cause autoinhibition that weakens DNA-binding affinity (37). D/E repeats are found in many DNA/RNA-binding proteins (50) and cause autoinhibition *via* intramolecular interactions with positively charged domains (37, 51, 52, 53, 54, 55, 56, 57, 58, 59). Because protein–DNA interactions involve extensive electrostatic interactions (60), the negatively charged DNA and the negatively charged C-terminal tail can compete for the positively charged DNA-binding domains (*i.e.*, A-box and B-box). The proteolytic removal of the D/E repeats would eliminate the autoinhibition and promote the binding activities of HMGB1.

**Figure 5:**
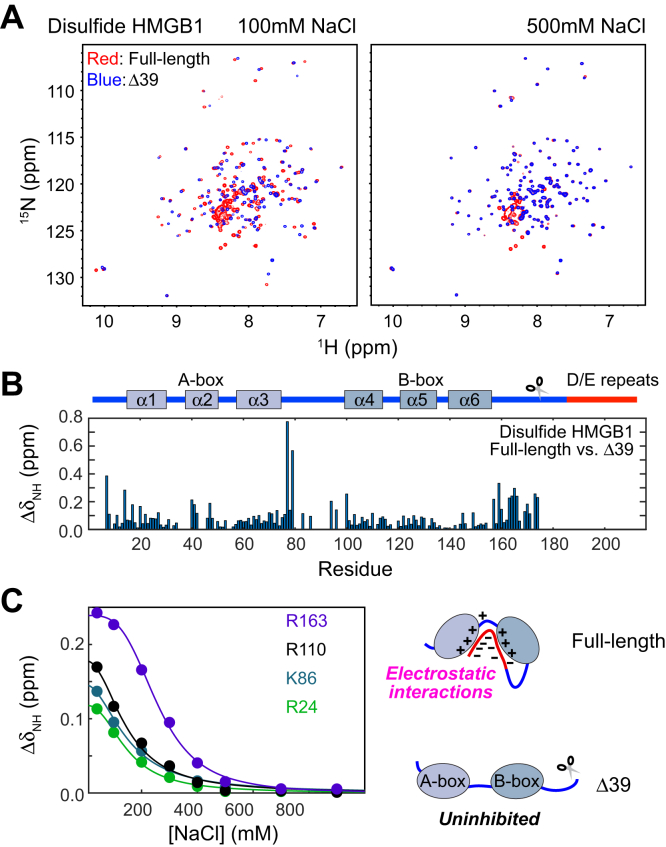
**Intramolecular interactions of disulfide HMGB1.***A*, overlaid ^1^H-^15^N TROSY spectra recorded for full-length disulfide HMGB1 (*red*) and the Δ39 product (*blue*) at 100 and 500 mM NaCl. Note that the two spectra are notably different at 100 mM NaCl but remarkably similar at 500 m NaCl. *B*, NMR chemical shift differences between the full-length disulfide HMGB1 and the Δ39 proteins for individual residues at 100 mM NaCl. These differences suggest the interactions between the C-terminal region and other regions in the full-length disulfide HMGB1 protein. *C*, the NMR chemical shift differences between the full-length HMGB1 and Δ39 proteins at various NaCl concentrations. Due to their electrostatic nature, the intramolecular interactions within the full-length protein are disrupted at high concentrations of NaCl, causing NMR chemical shifts that are virtually identical to those of the Δ39 product.

To examine whether disulfide HMGB1 also undergoes the same intramolecular interactions, we conducted NMR experiments on ^15^N-labeled FL and Δ39 proteins of disulfide HMGB1. Compared to all-thiol HMGB1, disulfide HMGB1 exhibited different NMR chemical shifts only for the A-box domain, suggesting that the disulfide bond in the A-box domain does not influence the other parts of the protein (Fig. S6 in the Supporting information). Figure 5*A* shows an overlay of the ^1^H-^15^N heteronuclear correlation spectra recorded for the FL and Δ39 proteins at 100 mM and 500 mM NaCl. At 100 mM NaCl, many residues exhibited significantly different NMR chemical shifts for the FL and Δ39 proteins (Fig. 5*B*). However, as the concentration of NaCl was increased, the differences in NMR chemical shifts became smaller (Fig. 5*C*). When the NaCl concentration was 500 mM or higher, the two proteins exhibited virtually identical chemical shifts. These results suggest that the negatively charged D/E repeats electrostatically interact with the positively charged parts in the FL protein of disulfide HMGB1 at physiological ionic strength.

The intramolecular interaction between the C-terminal tail and the DNA-binding domains might also make the cleavage site less accessible for neutrophil elastase. When HMGB1 binds to DNA, the C-terminal tail is displaced from the DNA-binding domains. This displacement may make the cleavage site more accessible and help accelerate the proteolytic processing of HMGB1 by neutrophil elastase.

## Discussion

Our current study demonstrates that DNA mediates the proteolytic processing of HMGB1 by neutrophil elastase and makes the cleavage more efficient and specific. Importantly, this proteolytic processing enhances binding affinities of disulfide HMGB1 for its extracellular targets. The affinity enhancement arises from the proteolytic removal of the segment containing the D/E repeats (residues 186–215) that cause dynamic autoinhibition. The binding affinity enhancement as well as the removal of the regulatory parts may play a role in innate immune response involving HMGB1.

**Figure 6:**
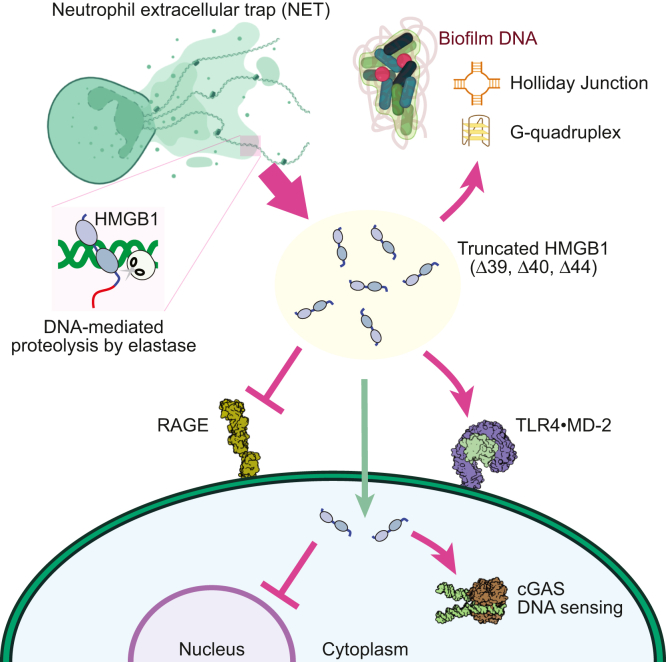
**Potential roles of the DNA-mediated proteolytic processing of HMGB1 by neutrophil elastase in NETs.** Due to the enhanced binding activities of the processed HMGB1 protein, this processing may promote (1) TLR4 signaling, (2) binding to biofilm DNA, and (3) DNA sensing by cGAS. Due to the loss of residues 177–215, the processing of HMGB1 may diminish (4) RAGE signaling and (5) nuclear localization. NET, neutrophil extracellular trap.

Figure 6 schematically summarizes potential roles of the DNA-mediated proteolytic processing of HMGB1 by neutrophil elastase in NETs. The segment removed from HMGB1 by neutrophil elastase contains not only the D/E repeats but also the second nuclear localization signal (NLS2; residues 179–185) and a part of the putative binding site for the receptor for advanced glycosylation endproducts (RAGE). Therefore, it is likely that the HMGB1 truncation diminishes the interaction with RAGE as well as translocation into nuclei. The enhancement of HMGB1’s binding affinities for TLR4 may increase the potency of HMGB1 as a DAMP that warns other cells. Considering that HMGB1 is involved in DNA uptake by some cells (15, 16, 17), it seems possible that the increase in DNA-binding affinity of HMGB1 promotes endocytosis of extracellular DNA. This may facilitate detection of pathogen’s DNA (*e.g.*, scaffold DNA in bacterial biofilm) by cytosolic DNA sensors involved in innate immunity. Production of cyclic GMP-AMP by the cytosolic DNA sensor cGAS requires formation of DNA fragment ladders, which HMGB1 facilitates *via* its ability to bend DNA (23). It should be noted that the removal of the C-terminal tail from HMGB1 causes more efficient DNA bending (61). Therefore, the DNA-mediated proteolysis of HMGB1 by neutrophil elastase might also promote cGAS activation.

The findings in our current work implicate that HMGB1 and neutrophil elastase may synergistically strengthen their proinflammatory effects. This is important because both neutrophil elastase and HMGB1 have been regarded as therapeutic targets for many inflammatory disorders (62, 63, 64). If hyperactivation of HMGB1 through the proteolytic processing by neutrophil elastase causes adverse inflammatory response, inhibition of neutrophil elastase may be a reasonable strategy to mitigate HMGB1’s adverse effect. In an early stage of inflammatory disorders, inhibition of HMGB1 might also be effective because extracellular HMGB1 stimulates neutrophils and causes them to produce NETs involving neutrophil elastase (24, 25). Cell biological studies would provide more insights into the relevance of DNA-mediated proteolysis of HMGB1 by neutrophil elastase to the inflammatory disorders.

## Experimental procedures

### Reagents

Chemicals and reagents were purchased from Sigma–Aldrich unless indicated otherwise. Lyophilized human neutrophil elastase (CAS 9004-06-2) was purchased from Sigma–Aldrich (catalog no.: #324681). Lyophilized TLR4•MD-2 complex was purchased from R&D Systems (catalog no.: #3146-TM-050). All chemically synthesized DNA strands were purchased from Integrated DNA Technologies. A HPLC-purified 32-mer strand of 5′-fluorescein amidite (FAM)–labeled G-quadruplex DNA with a sequence of AGGGCGGTGTGGGAAGAGGGAAGAGGGGGAGG, which corresponds to “FAM-32G” in Amato *et al.* (42), was purchased from Integrated DNA Technologies. The individual strands of the linear 20 bp DNA duplex with a sequence, CTCTGGACCTTCCTTTCTTC, were purchased from Integrated DNA Technologies and purified using Mono-Q anion exchange chromatography. The FAM-labeled Holliday junction DNA J1 was composed of four oligonucleotides designed by Kallenbach *et al.* (43): J1A, CGCAATCCTGAGCACG; J1B, CGTGCTCA CCGAATGC; J1C, GCATTCGGACTATGGC; and J1D, GCCATAGTGGATTGCG. The FAM moiety was attached to the 5′ terminus of the J1A strand during DNA synthesis. Each strand was purified using Mono-Q anion exchange chromatography. After annealing of an equimolar mixture of the four strands, the Holliday junction DNA J1 was purified by Mono-Q anion exchange chromatography. The ultrapure calf thymus DNA solutions were purchased from Invitrogen (catalog no.: #15633019) and were sonicated to produce DNA fragments with an average length of ∼500 bp.

### Preparation of disulfide HMGB1 and its truncated variants

Recombinant HMGB1 was expressed in *Escherichia coli* strain BL21(DE3) using a plasmid encoding the amino-acid sequence of GenBank CAG33144.1. The protein was purified from lysates using SP-FF cation-exchange, Resource-Q anion-exchange, and Sephacryl S-100 HR size-exclusion columns (GE Healthcare), as previously described (37). For the NMR analysis, ^15^N- or ^13^C/^15^N-labeled proteins were expressed in minimal media containing ^15^NH_4_Cl (Cambridge Isotope Laboratories) and ^13^C glucose (Cambridge Isotope Laboratories) as the sole nitrogen and carbon sources, respectively. The plasmids encoding the truncated HMGB1 variants (Δ39, Δ40, and Δ44) were prepared using a Quick-Change Lightening mutagenesis kit (Agilent) by introducing a stop codon at K177, V176, or K172. The truncated variants were expressed in *Escherichia coli* strain BL21(DE3) and purified using SP-FF cation-exchange, Resource S cation-exchange, and Sephacryl S-100 HR size-exclusion columns, as previously described for the Δ30 variant (37).

### Kinetic assays of HMGB1 proteolysis by neutrophil elastase

A 50 μg of lyophilized neutrophil elastase was dissolved in a 175 μl buffer of 50 mM sodium acetate (pH 5.5) with 200 mM NaCl. The solution was divided into small aliquots (∼10 μl), immediately frozen, and kept at −70 °C until use. The reaction for the proteolysis of disulfide HMGB1 by neutrophil elastase in the presence and absence of the linear 20 bp DNA duplex (20 μM) or sonicated calf thymus DNA (400 μM base pairs) was conducted at a room temperature in a buffer of 10 mM potassium phosphate (pH 7.4) and 100 mM NaCl. The concentration of neutrophil elastase was 20 nM. SDS-PAGE was conducted with a Novex 4% to 20% polyacrylamide gel (Invitrogen) and stained with GenScript eStain L1 protein staining system. Each gel image was converted into gray scale and analyzed with ImageJ software (https://imagej.nih.gov/ij) (65) for densitometry. The apparent rate constants *k*_*app*_ in s^−1^ units were determined from the time course of the population of the intact HMGB1 protein. The reaction rates as the initial velocity (36) were calculated as *k*_*app*_[S]_0_, where [S]_0_ is the initial substrate concentration (*i.e.*, the total concentration of disulfide HMGB1). The parameters *K*_*m*_ and *k*_cat_ were determined through the nonlinear least squares fitting to the reaction rate data with the Michaelis–Menten equation.

### Mass spectrometry for identifying the HMGB1 proteolysis sites

To identify the proteolysis sites, LC-MS intact mass analysis was conducted for control samples and the reaction mixtures of disulfide HMGB1 cleaved by neutrophil elastase in the presence or absence of the 20 bp DNA duplex under the conditions described above. The reaction was stopped by freezing and kept at −20 °C until ready for use. Immediately after thawing the reaction mixtures, samples were diluted 1:1 with a solution of 50% acetonitrile and 0.2% formic acid and analyzed by LC-MS to determine the intact mass profile of each sample. LC-MS intact mass analysis was accomplished using a desalting size-exclusion chromatography column (BEH SEC200 4.6 × 30 mm, Waters) and a denaturing isocratic mobile phase (30% acetonitrile, 0.1% formic acid, 0.02% TFA) controlled by a Vanquish Horizon UHPLC (Thermo Scientific) with the eluent coupled directly to an Orbitrap Eclipse MS system (Thermo Scientific), using charge reduction of the eluted intact protein ions to improve spectral fidelity as previously reported (66). Raw LC-MS data were deconvolved and annotated using BioPharma Finder software (version 4.1, Thermo Scientific). Deconvolved masses were annotated using a maximum mass tolerance of 100 ppm relative to the theoretical mass of each identified species.

### DNA-binding affinity measurements

Binding affinities of HMGB1 and its variants for quadruplex DNA 32G or Holliday junction J1 were measured using FAM fluorescence anisotropy. FAM fluorescence anisotropy was measured with an ISS PC-1 spectrofluorometer using an excitation wavelength of 490 nm and an emission wavelength of 521 nm at 25 °C. The binding assays for FAM-32G were conducted in a buffer of 10 mM potassium phosphate (pH 7.4) and 200 mM NaCl. The concentration of FAM-32G was 4 nM. The binding assays for FAM-labeled Holliday junction was conducted in a buffer of 10 mM potassium phosphate (pH 7.4) and 150 mM NaCl. The concentration of FAM-Holliday junction was 4 nM. The dissociation constant *K*_*D*_ was determined from the anisotropy data using MATLAB (MathWorks, Inc), as previously described (67). The measurements of each dissociation constant *K*_*d*_ were triplicated. The statistical significance was assessed with a *p* value from a *t* test when the data were compared for the FL and truncation variant HMGB1 proteins.

### HMGB1–TLR4•MD-2 binding assays

SPR-based assays for the binding of disulfide HMGB1 to TLR4•MD-2 complex was conducted using a GE Healthcare Biacore T-100, essentially in the same manner as described by He *et al.* (49). A 50 μg of lyophilized TLR4•MD-2 complex was dissolved in a 0.5 ml buffer of PBS at pH 7.4 and stored in aliquots at −70 °C until use. A 1.1 μM solution of TLR4•MD-2 complex in 10 mM sodium acetate buffer (pH 4.5) was prepared immediately before immobilization. The complex was immobilized onto a CM5 sensor chip (Cytiva) using an amine coupling kit (Cytiva) for covalent attachment to the sensor surface. The reference flow cell and the ligand flow cell were activated with a 1:1 mixture on N-hydroxysuccinimide and N-ethyl-N-dimethylaminopropyl-carbodiimide. The solution of TLR4•MD-2 complex was injected at a flow rate of 10 μl/min and was stopped when the SPR reached 1200 response units. The SPR assays for the interaction between HMGB1 and TLR4•MD-2 complex were conducted in a buffer of 10 mM Hepes (pH 7.4), 150 mM NaCl, 3 mM EDTA, and 0.005% v/v Tween-20. The protein concentrations were 31.25, 62.5, 125, 250, 500, and 1000 nM for the FL disulfide HMGB1 protein and 1.0, 1.5, 3.1, 6.2, 12.5, and 16.6 nM for the Δ39 product. The protein solutions were injected at a flow rate of 30 μl/min at 25 °C. The duration for the association phase was set to 60 s and the duration for the dissociation phase was set for 120 s. Three independent experiments were performed. The dissociation constant (*K*_*d*_), the association rate constant (*k*_*on*_), and the dissociation rate constant (*k*_*off*_) were determined along with a 1:1 binding model using the Biacore T100 Evaluation software 2.0.4 (GE Healthcare). For each HMGB1 construct, the SPR measurements were triplicated. The statistical significance was assessed with a *p* value from a *t* test when the data were compared for the FL and truncation variant HMGB1 proteins.

### NMR experiments on intramolecular interactions of disulfide HMGB1

The Cys23-Cys45 disulfide bond was confirmed with NMR spectra, as previously described (35). The intramolecular interactions between the C-terminal D/E repeats and A-/B-box domains were investigated through analysis of NMR chemical shift differences between the FL HMGB1 and Δ39 variant. The resonances for the oxidized proteins were assigned using 3D HNCA, HN(CO)CA, HNCACB, CBCA(CO)NH, CC(CO)NH, HNCO, and HN(CA)CO spectra (68). The NMR experiments were conducted using a Bruker Avance III spectrometer operated at the ^1^H frequency of 750 MHz. The NMR data were processed with NMR-Pipe program (69) and the spectra were analyzed with the NMRFAM-SPARKY program (70). The resonance assignment data for the FL and truncated variant disulfide HMGB1 proteins were deposited to Biological Magnetic Resonance Data Bank (BMRB) (accession codes, 51641 and 51640, respectively). Salt titration samples were made of 270 μl solutions with 0.3 mM ^15^N-labeled FL protein or Δ39 variant, in a buffer containing 40 to 900 mM NaCl, 10 mM potassium phosphate (pH 7.5), and 1 mM sodium 2,2-dimethyl 2-silapentane-5-sulfonate (DSS). Eight different concentrations of NaCl (40, 100, 200, 300, 400, 500, 700, and 900 mM) were used. For each, a ∼270 μl solution was sealed in an inner tube (the diameter 3.2 mm) with a 100 μl of D_2_O in the outer tube of Shigemi coaxial NMR tubes (the diameter 5.0 mm). The coaxial tubes were used to achieve optimal impedance matching for the cryogenic probe on high ionic strength samples (71). For each sample, 1D ^1^H and ^1^H-^15^N transverse relaxation optimized spectroscopy (72) spectra were recorded at 25 °C. NMR chemical shifts were referenced to the ^1^H signal from DSS as the internal reference. For each backbone NH group, a unified chemical shift difference $\Delta \delta ={\left[{\left(\delta_{H}^{FL}-\delta_{H}^{\Delta 30}\right)}^{2}+0.25{\left(\delta_{N}^{FL}-\delta_{N}^{\Delta 30}\right)}^{2}\right]}^{1/2}$, where *δ*_*H*_ and *δ*_*N*_ represent ^1^H and ^15^N chemical shifts, was calculated from the resonances of the FL and Δ39 proteins at each concentration of NaCl. Fittings to the NaCl concentration-dependent Δ*δ* data were conducted using MATLAB software, as previously described (37).

## Data availability

All data are contained within the main text or the Supporting information.

## Supporting information

This article contains supporting information.

## Conflict of interest

The authors declare that they have no conflict of interest with the contexts of this article.
