# Supplementary material for: DNA-mediated proteolysis by neutrophil elastase enhances binding activities of the HMGB1 protein
Source: J Biol Chem. 2022 Oct 8;298(11):102577. doi: 10.1016/j.jbc.2022.102577 (PMC9664404; doi:10.1016/j.jbc.2022.102577)
Supplement: Supplemental Figures S1–S6 and Table S1 [file mmc1.pdf]

SUPPORTING INFORMATION

DNA-mediated proteolysis by neutrophil elastase enhances binding activities of the HMGB1 protein

Xi Wang, Marlen Mayorga-Flores, Karina G. Bien, Aaron O. Bailey, and Junji Iwahara*

Contents

Fig. S1: Pictures of the SDS-PAGE gels used for Figure 1A

Fig. S2: Locations of the active site and the positively charged surface of neutrophil elastase

Fig. S3: Impact of the D/E repeats on HMGB1 cleavage by neutrophil elastase

Fig. S4: Deconvoluted mass spectra recorded for the HMGB1 and Δ40 samples as control data

Fig. S5: G-quadruplex binding isotherm data for the Δ40 and Δ44 products of disulfide HMGB1

Fig. S6: Differences in NMR chemical shifts between all-thiol and disulfide HMGB1 proteins

Table S1: Summary of LC-MS data on the proteolysis of HMGB1 by neutrophil elastase

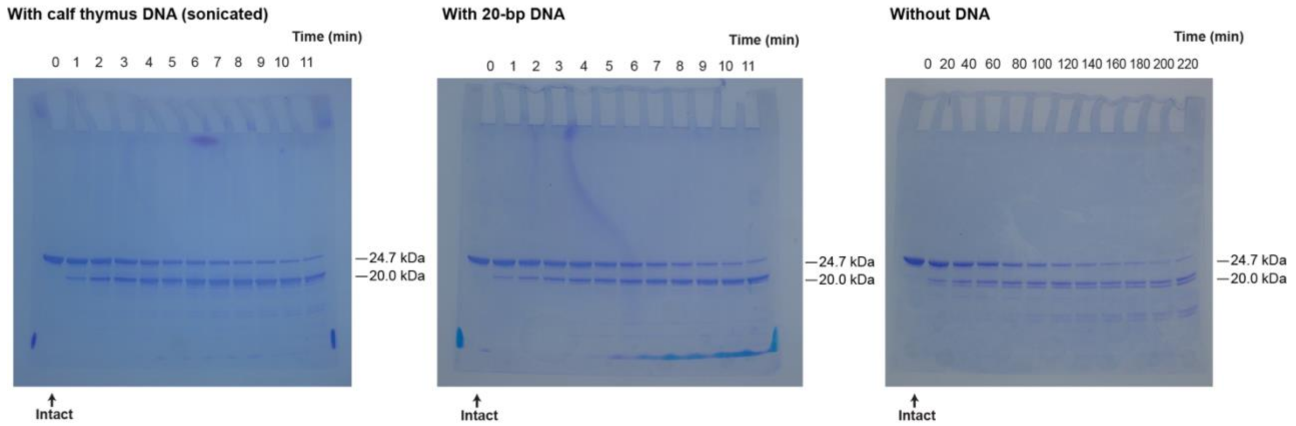

Fig. S1. Pictures of the SDS-PAGE gels used for 1A. The original color pictures are shown, although the images converted into gray scale were used for the densitometric analysis. The conditions for the reaction in the presence of DNA were 10 μM HMGB1, 20 nM neutrophil elastase, 400 μM bp sonicated calf thymus DNA (average length, ~0.5 kbp), or 20 μM 20-bp DNA (with a sequence of CTCTGGACCTTCCTTTCTTC) in a buffer of 10 mM potassium phosphate (pH 7.4) and 100 mM NaCl. The conditions for the reaction in the absence of DNA were the same except that no DNA was used. The indicated molecular weights are based on the mass spectrometry data (see Figure 2 and Table S1). Note that the reaction in the absence of DNA showed additional products other than Δ39, Δ40, and Δ44 over ~2-4 hours. Oval blobs in the gel picture for the reaction in the presence of DNA are air bubbles between the gel and the illuminator surface and did not interfere with the densitometric analysis.

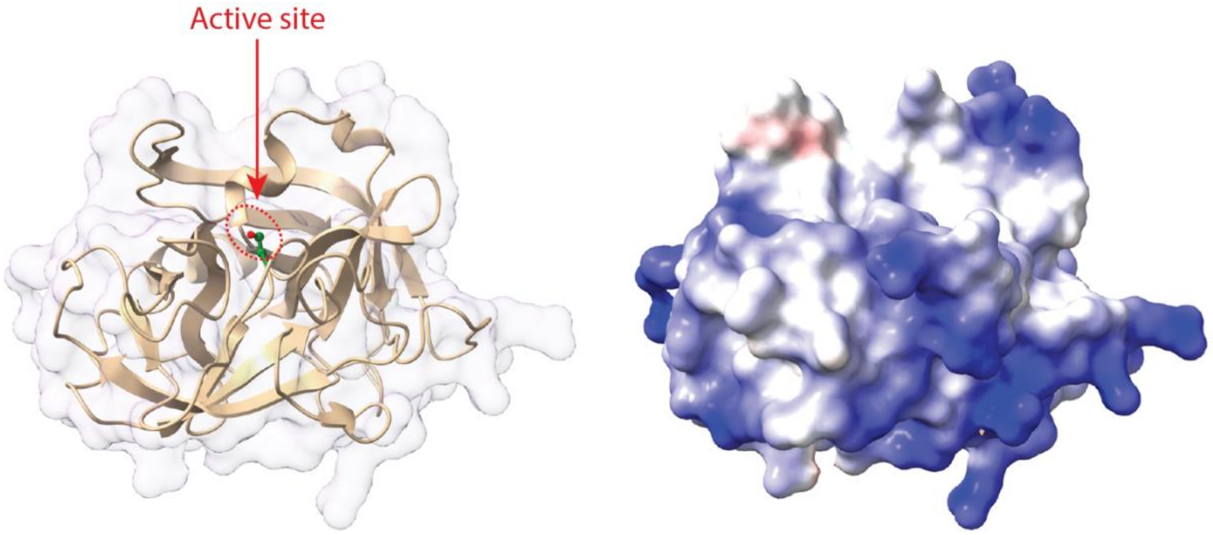

Fig. S2. Locations of the active site and the positively charged surface in a 1.86-Å resolution crystal structure of neutrophil elastase (PDB 3Q76). The surface electrostatic potentials are colored in blue (positive) and red (negative).

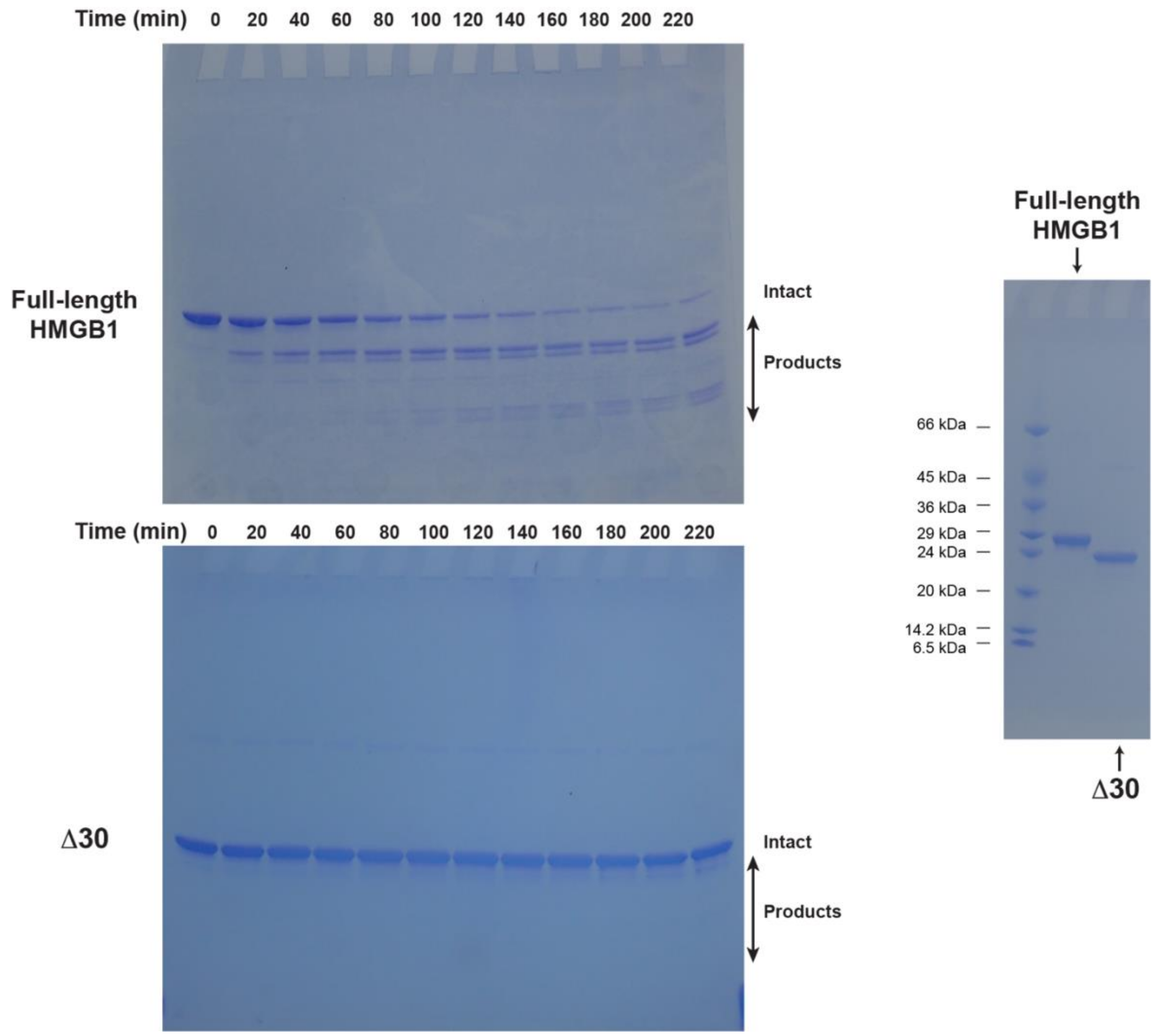

Fig. S3: Impact of the D/E repeats on HMGB1 cleavage by neutrophil elastase in the absence of DNA. The SDS-PAGE gels shown on the left-hand side show the time courses of the proteolysis of the full-length disulfide HMGB1 (top) or its Δ30 variant (bottom), which lacks the D/E repeats but retains the cleavage site. The gel picture for the full-length disulfide HMGB1 (top) is identical to the one shown in Fig. S1 for "without DNA". The conditions for the reaction were 10 μM HMGB1, 20 nM neutrophil elastase in a buffer of 10 mM potassium phosphate (pH 7.4) and 100 mM NaCl. The SDS-PAGE gel shown on the right-hand side is for the intact full-length disulfide HMGB1 and Δ30 variant along with molecular weight markers.

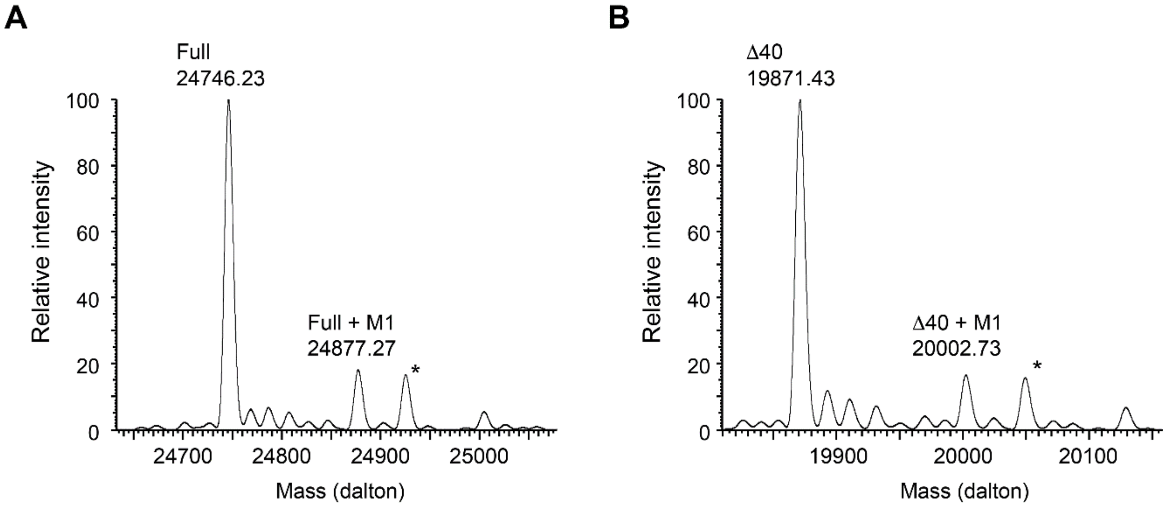

Fig. S4. Deconvolved mass spectra recorded for the full-length disulfide HMGB1 and Δ40 samples as control data. These proteins were expressed in *E. coli* and purified by chromatographic methods as described in the Experimental Procedures section of the main text. The highest peaks were observed at the mass values expected for the proteins lacking the initial methionine M1, presumably due to *E. coli* peptidase. Minor peaks were observed at the mass values expected for the proteins retaining M1 (indicated by "Full + M1" and "Δ40 + M1") and the gluconoylated proteins lacking M1 (indicated by asterisks).

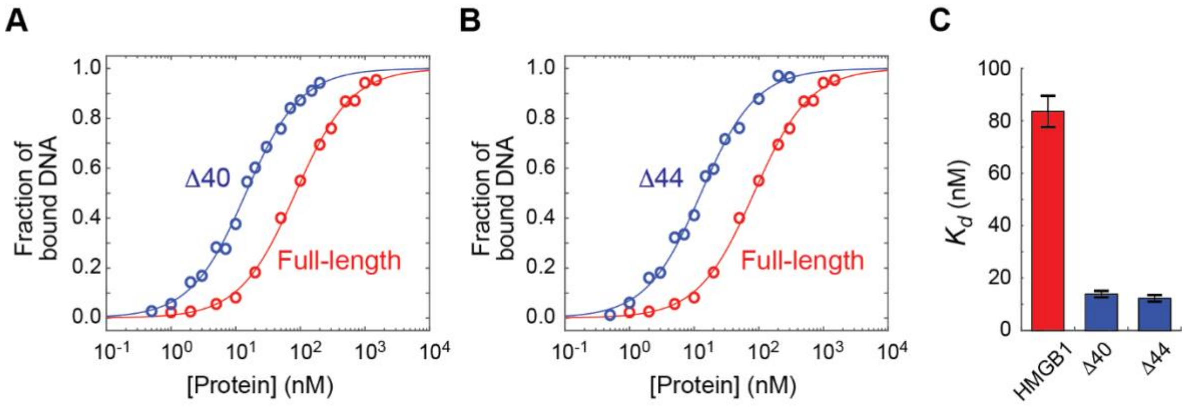

Fig. S5. G-quadruplex binding isotherm data for the Δ40 and Δ44 products of disulfide HMGB1. (**A, B**) Fluorescence anisotropy-based binding assay data used to measure the affinity of the Δ40 and Δ44 products for the DNA G-quadruplex FAM-32G. The data for the full-length disulfide HMGB1 protein (red) are also shown for comparison. The experimental conditions are the same as those used for Figure 3A in the main text. (**C**) Dissociation constants K_d for the full-length disulfide HMGB1, Δ40, and Δ44 proteins.

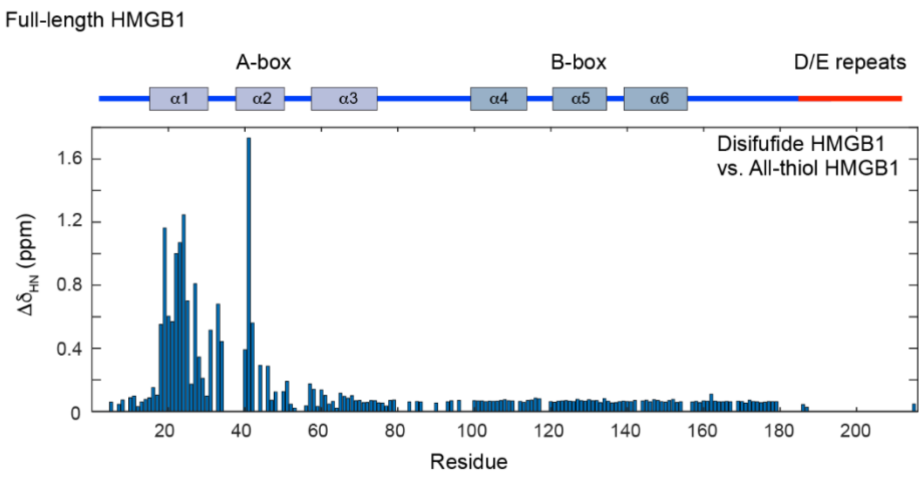

Fig. S6. Differences in NMR chemical shifts between all-thiol and disulfide HMGB1 proteins. The disulfide bond between Cys23 and Cys45 causes large changes in NMR chemical shifts in the A-box domain but does not impact the other parts of HMGB1.

Table S1. Summary of LC-MS data on the species identified in the reaction mixtures of neutrophil elastase proteolysis of HMGB1.[a]

Sequence name [b]	Residue range	Modification [c]	Average mass	Theoretical mass	Error (ppm)	Sum intensity	Relative abundance	Fractional abundance
**Cleavage reaction in the presence of DNA**								
Δ39	2-176		19970.67	19970.91	12.1	6.32E+08	100.00	23.69
Δ40	2-175		19871.05	19871.78	36.7	2.61E+08	41.21	9.76
Δ44	2-171		19458.79	19459.25	23.5	1.33E+08	21.08	4.99
Δ39 + M1	1-176		20101.43	20102.11	33.6	9.07E+07	14.35	3.40
Full	2-215		24745.47	24746.36	35.9	5.42E+07	8.58	2.03
Δ39	2-176	1 gluconoylation	20149.53	20149.05	24.0	4.33E+07	6.84	1.62
Δ40 + M1	1-175		20002.99	20002.97	0.7	2.33E+07	3.69	0.87
Δ39	2-176	1 phosphogluconoylation	20228.76	20229.03	13.4	1.39E+07	2.19	0.52
Δ44 + M1	1-175		19588.81	19590.44	83.3	1.28E+07	2.03	0.48
Full	2-215	1 gluconoylation	24924.12	24924.50	15.3	1.17E+07	1.86	0.44
**Cleavage reaction in the absence of DNA**								
Sequence name [b]	Residue range	Modification [c]	Average mass	Theoretical mass	Error (ppm)	Sum intensity	Relative abundance	Fractional abundance
Full	2-215		24746.14	24746.36	8.7	3.78E+09	100.00	36.56
Full + M1	1-215		24876.88	24877.56	27.1	6.05E+08	16.00	5.85
Δ39	2-176		19970.57	19970.91	17.1	6.01E+08	15.90	5.81
Δ40	2-175		19871.17	19871.78	30.6	5.68E+08	15.01	5.49
Full	2-215	1 gluconoylation	24925.13	24924.50	25.1	5.02E+08	13.28	4.86
Δ44	2-171		19459.01	19459.25	12.4	3.24E+08	8.58	3.14
Full	2-215	1 phosphogluconoylation	25004.75	25004.48	10.9	1.61E+08	4.26	1.56
Δ39	2-176	1 gluconoylation	20150.30	20149.05	61.8	1.29E+08	3.41	1.25
Δ39 + M1	1-176		20101.71	20102.11	19.7	8.98E+07	2.37	0.87
Δ40 + M1	1-175		20003.37	20002.97	19.9	4.69E+07	1.24	0.45
Δ40	2-175	1 gluconoylation	20050.78	20049.92	43.1	4.17E+07	1.10	0.40

[a] The amino-acid sequence of HMGB1 is from GenBank CAG33144.1.

[b] These names are indicated in Figures 2 and S2.

[c] Modification other than disulfide bond. All species have a disulfide bond.
